# Proteasomal turnover of the RhoGAP tumor suppressor DLC1 is regulated by HECTD1 and USP7

**DOI:** 10.1038/s41598-022-08844-3

**Published:** 2022-03-23

**Authors:** Yannick Frey, Mirita Franz-Wachtel, Boris Macek, Monilola A. Olayioye

**Affiliations:** 1 Institute of Cell Biology and Immunology, University of Stuttgart, Allmandring 31, 70569 Stuttgart, Germany; 2 Proteome Center Tübingen, University of Tübingen, Auf der Morgenstelle 15, 72076 Tübingen, Germany; 3 Stuttgart Research Center Systems Biology (SRCSB), University of Stuttgart, 70569 Stuttgart, Germany

**Keywords:** RHO signalling, Focal adhesion, Proteolysis

## Abstract

The Rho GTPase activating protein Deleted in Liver Cancer 1 (DLC1) is frequently downregulated through genetic and epigenetic mechanisms in various malignancies, leading to aberrant Rho GTPase signaling and thus facilitating cancer progression. Here we show that in breast cancer cells, dysregulation of DLC1 expression occurs at the protein level through rapid degradation via the ubiquitin–proteasome system. Using mass spectrometry, we identify two novel DLC1 interaction partners, the ubiquitin-ligase HECTD1 and the deubiquitinating enzyme ubiquitin-specific-processing protease 7 (USP7). While DLC1 protein expression was rapidly downregulated upon pharmacological inhibition of USP7, siRNA-mediated knockdown of HECTD1 increased DLC1 protein levels and impaired its degradation. Immunofluorescence microscopy analyses revealed that the modulation of HECTD1 levels and USP7 activity altered DLC1 abundance at focal adhesions, its primary site of action. Thus, we propose opposing regulatory mechanisms of DLC1 protein homeostasis by USP7 and HECTD1, which could open up strategies to counteract downregulation and restore DLC1 expression in cancer.

## Introduction

As key regulators of cytoskeleton remodeling, Rho GTPases are required for diverse biological processes ranging from cell morphology and polarity, organelle positioning and membrane transport, cell division and motility. Rho GTPase activation is brought about by guanine nucleotide exchange factors (GEFs) that facilitate exchange of GDP for GTP, inducing the binding and activation of downstream effector pathways. By contrast, GTPase-activating proteins (GAPs) enhance the low intrinsic GTPase activity of Rho proteins to return them into the inactive, GDP-bound state and terminate the signal^1,2^. During cancer progression, aberrant activity of Rho proteins contributes to loss of cell polarity and cell–cell contacts as well as acquisition of a more motile phenotype, which enables epithelial cells to invade neighboring tissues. Dysregulation of Rho signaling has mainly been attributed to Rho GTPase overexpression or altered regulation by GEFs and GAPs^3,4^. In this regard, the RhoGAP Deleted in Liver Cancer 1 (*DLC1*) has been established as a bona fide tumor suppressor, characterized by frequent copy number loss or transcriptional silencing in different tumor entities^5^. In various cellular model systems, DLC1 suppressed cell proliferation, cell migration and invasion as well as clonogenicity^6–9^. On the molecular level, the tumor suppressive function of DLC1 has been mainly ascribed to its RhoGAP activity. Through the binding of tensins, talin and focal adhesion kinase (FAK) DLC1 is recruited to focal adhesions (FAs) where it is thought to locally restrict RhoA signaling^10,11^. Thus, DLC1 can be functionally inactivated by missense mutations in the coding sequence or post-translational modifications (PTMs) which interfere with protein interactions and result in altered subcellular localization^11–15^.

Opposed to the regulation of DLC1 activity by phosphorylation, the regulation of DLC1 proteostasis is less well understood. A better understanding of the mechanisms of DLC1 protein degradation could provide strategies to restore its expression in malignancies. Protein degradation is most commonly mediated by the ubiquitin–proteasome-system, whereby addition of multiple moieties of the small protein ubiquitin (Ub) to specific lysine residues of target proteins by an E3 ubiquitin ligase of the HECT or RING family serves as recognition signal for the proteasomal degradation machinery^16^. Ubiquitination is counteracted by the protease family of deubiquitinating enzymes (DUBs). The aberrant activity of either E3 ligases or DUBs is known to contribute to dysregulated protein homeostasis during cancer development^17^. In non-small cell lung cancer (NSCLC), it was described that DLC1 can be ubiquitinated and subsequently degraded by cullin 4A-RING ubiquitin ligase (CRL4A) complex interaction with DDB1 and the FBXW5 substrate receptor^18^. However, it is unclear how and to what extent functional inactivation of DLC1 is achieved by degradation in other tumor entities. In breast cancer (BC), *DLC1* was found to be deleted in 33% of samples while promoter hypermethylation or missense mutations did not frequently occur^5,12,19^. Here, we investigate DLC1 proteasomal degradation in BC cell lines and uncover novel regulators of DLC1 protein stability.

## Results

### DLC1 is subject to rapid proteasomal degradation

**Figure 1:**
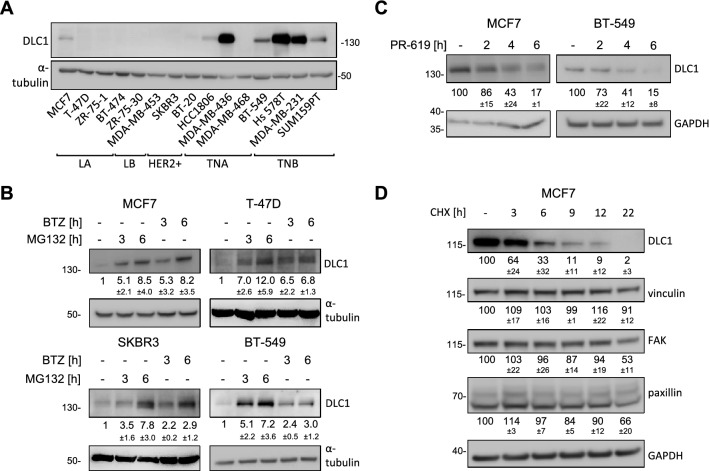
DLC1 undergoes rapid proteasomal degradation in breast cancer cells. (**A**) Lysates of the indicated breast cancer cell lines from different subgroups (luminal A (LA), luminal B (LB), HER2 positive (HER2 +), triple negative A (TNA), triple negative B (TNB)) were analyzed by immunoblotting with the indicated antibodies. (**B**) Cells were treated with MG-132 (10 µM) or bortezomib (BTZ, 100 ng/ml) as indicated and the lysates were analyzed by immunoblotting with the indicated antibodies. Control sample was treated with DMSO for 6 h. Western blots from three independent experiments were analyzed with ImageJ, the fold change in DLC1 expression was determined by normalizing the DLC1/α-tubulin ratio to that of control sample and is presented as mean ± s.d. Cropped blots shown from different cell lines are derived from separate blots. (**C**) Cells were treated with PR-619 (20 µM) as indicated and the lysates were analyzed by immunoblotting with the indicated antibodies. The control sample was treated with DMSO for 6 h. Western blots from three independent experiments were analyzed with ImageJ, the fold change in DLC1 expression was determined by normalizing the DLC1/GAPDH ratio to that of control sample and is presented as mean ± s.d. Cropped blots shown from different cell lines are derived from separate blots. (**D**) MCF7 cells were treated with cycloheximide (CHX, 60 µg/ml) and lysates were analyzed by immunoblotting with the indicated antibodies. Western blots from three independent experiments were analyzed with ImageJ, the fold change in protein expression was determined by normalizing the signal to GAPDH and to the control sample and is presented as mean ± s.d. (**A**, **B**, **C**, **D**) All western blots shown are representative of three independent experiments. Full-length western blots are provided as Supplementary Information.

We first analyzed the expression levels of the DLC1 protein in a panel of BC cell lines of various subtypes (Fig. 1A). While in some triple-negative breast cancer cell lines DLC1 was robustly expressed most cell lines of the luminal A/B, HER2+ , and also TNA and TNB subtype showed low or barely detectable DLC1 protein expression. To explore whether protein degradation might be a contributing factor to the low DLC1 expression, we treated cells with the proteasome inhibitors bortezomib (BTZ) and MG-132. Indeed, DLC1 protein levels were markedly increased after short time treatment with the proteasome inhibitors bortezomib (BTZ) and MG-132 (Fig. 1B). Notably, expression was also restored in T-47D and SKBR3 cell lines previously described as DLC1-negative^14,20^. The reduction of DLC1 levels in cells treated with the broad-spectrum DUB inhibitor PR-619 confirmed the involvement of the ubiquitin–proteasome system in DLC1 degradation (Fig. 1C), as DLC1 mRNA remained stable (Supplementary Figure S1). Further, compared to other FA-associated proteins, DLC1 showed a shorter protein half-life in cycloheximide chase assays (Fig. 1D), indicating that rapid DLC1 degradation is not simply linked to general high turnover rates of FAs.

To identify regulating molecular factors of DLC1 degradation, we performed mass spectrometry analysis of DLC1-interacting proteins. GFP-tagged wild-type DLC1 was transiently expressed in MCF7 cells and immunopurified from cell lysates, followed by analysis of the samples by nano-liquid chromatography tandem mass spectrometry (nanoLC-MS/MS). In order to exclude the bulk of non-specific binders, we only considered proteins that associated with DLC1 and were not present in the empty vector control (Supplementary Table S1). Among these identified candidates, the already reported interaction partners EEF1A1, PP2A and different isoforms of 14–3-3 were included^13,21,22^. Interestingly, two proteins directly associated with ubiquitination were detected: the HECT family E3 ligase HECTD1 and the DUB ubiquitin-specific-processing protease 7 (USP7). HECTD1 has been described previously to regulate FA dynamics by governing the degradation of phosphatidylinositol 4-phosphate 5-kinase type I γ^23^. Besides its well-known role in stabilizing the ubiquitin ligase Mdm2, thus leading to degradation of the tumor suppressor p53, USP7 has been shown to regulate numerous other protein substrates^24^.

### HECTD1 and USP7 are novel regulators of DLC1 stability

**Figure 2:**
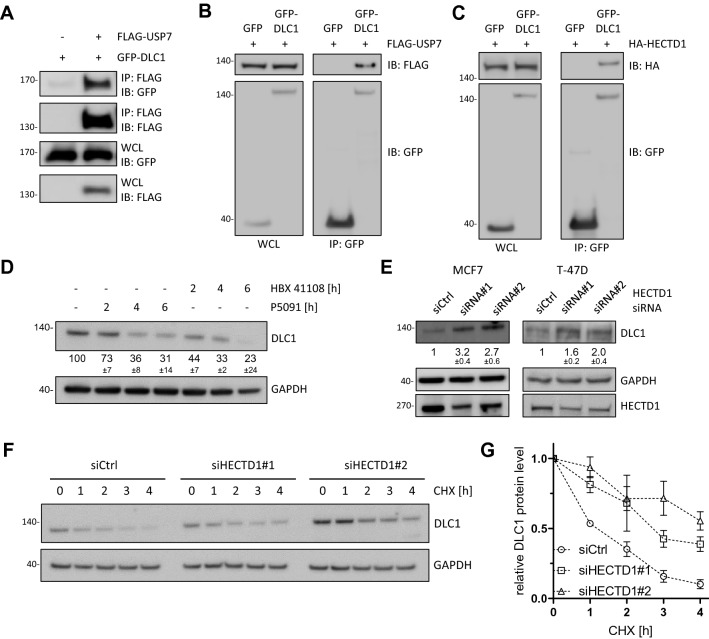
HECTD1 and USP7 are novel regulators of DLC1 stability. (**A**, **B**, **C**) HEK293T cells were transiently transfected with vectors encoding the indicated constructs or FLAG empty vector (−). (**A**) The next day, cells were lysed and immunoprecipitation with an anti-FLAG antibody was performed. Cell lysates (WCL) and precipitates were analyzed by immunoblotting using the indicated antibodies. Cropped blots showing co-immunoprecipitation signals are derived from blots with different exposure compared to WCL. (**B**, **C**) The next day, cells were lysed and immunoprecipitation with an anti-GFP-nanobody was performed. Cell lysates (WCL) and precipitates were analyzed by immunoblotting using the indicated antibodies. Cropped blots showing co-immunoprecipitation signals are derived from blots with different exposure compared to WCL. (**D**) MCF7 cells were treated with P5091 (20 µM) or HBX 41108 (10 µM) as indicated and lysates were analyzed by immunoblotting with the indicated antibodies. The control sample was treated with DMSO for 6 h. Western blots from three independent experiments were analyzed with ImageJ, the fold change in DLC1 expression was determined by normalizing the DLC1/GAPDH ratio to that of control sample and is presented as mean ± s.d. (**E**) Cells were transfected with control siRNA or two independent siRNAs targeting HECTD1. 72 h post transfection cells were lysed and lysates were analyzed by immunoblotting with the indicated antibodies. Cropped blots shown from different cell lines are derived from separate blots. Western blots from three independent experiments were analyzed with ImageJ, the fold change in DLC1 expression was determined by normalizing the DLC1/GAPDH ratio to that of control sample and is presented as mean ± s.d. (**F**) MCF7 cells were transfected with control siRNA or two siRNAs targeting HECTD1. 72 h post transfection cells were treated with cycloheximide (CHX, 60 µg/ml) as indicated and lysates were analyzed by immunoblotting with the indicated antibodies. (**G**) Quantification of protein levels from (**F**) by ImageJ. n = 4, error bars represent mean ± s.d. (**A**, **B**, **C**, **D**, **E**, **F**) Full-length western blots are provided as Supplementary Information.

To validate the interaction of DLC1 and USP7, FLAG-tagged USP7 was transiently expressed in HEK293T cells, immunoprecipitated from cell lysates and binding of co-expressed GFP-tagged DLC1 was confirmed by immunoblotting (Fig. 2A). Vice versa, FLAG-USP7 showed binding to GFP-DLC1, but not free GFP (Fig. 2B). Similarly, mouse HA-HECTD1, which shows 98% sequence identity to its human homologue, co-immunoprecipitated with GFP-tagged DLC1 (Fig. 2C). To investigate a potential role of the novel interaction partners in DLC1 degradation, MCF7 cells were treated with two different USP7 inhibitors, P5091 or HBX 41,108. Compared to treatment with the broad-spectrum DUB inhibitor PR-619 (Fig. 1C), DLC1 protein levels were decreased to a similar extent upon specific USP7 inhibition, indicating that USP7 might be the primary DUB responsible for DLC1 stabilization (Fig. 2D). qPCR analysis confirmed that the observed decrease in DLC1 abundance was not due to a decrease in DLC1 mRNA levels (Supplementary Figure S2). However, in TNBC cell lines expressing high levels of DLC1, no decreased expression was observed upon siRNA-mediated USP7 depletion (Supplementary Figure S3). Considering the vast number of proteins targeted by USP7^25^, the discrepancy between short-term inhibition of USP7 activity versus long-term depletion of the USP7 protein might stem from the integration of the regulatory effects of other USP7 substrates three days post transfection.

Because no specific inhibitors for HECTD1 ubiquitin-ligase activity are commercially available, we depleted its expression levels in MCF7 and T-47D cells using two independent specific siRNAs which manifested in increased DLC1 protein levels 3 days post transfection (Fig. 2E). However, for one of the two siRNAs we also noticed an increase of DLC1 mRNA levels (Supplementary Figure S4). To explore if the observed upregulation of DLC1 stems from impaired degradation, we performed cycloheximide-chase assays after HECTD1 knockdown in MCF7 cells (Fig. 2F, G). Albeit depletion of HECTD1 did not completely abrogate DLC1 degradation, the rate of degradation was noticeably slowed down. This may be explained by residual HECTD1 protein or other pathways involved in DLC1 turnover.

**Figure 3:**
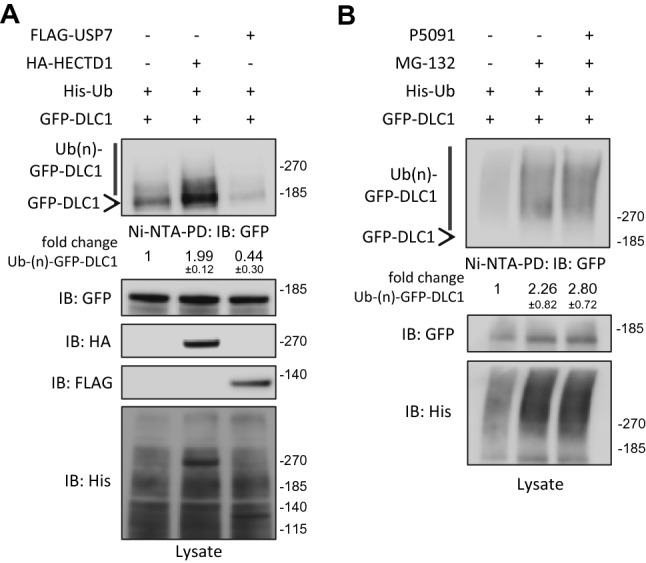
HECTD1 and USP7 regulate DLC1 ubiquitination. (**A**) HEK293T cells were transiently transfected with vectors encoding the indicated constructs. The next day, cells were treated with the proteasome inhibitor MG-132 (10 µM) before lysis. Lysates were subjected to pulldown with Ni–NTA-agarose. Pulldowns and lysates were analyzed by immunoblotting with the indicated antibodies. (**B**) MCF7 cells were transiently transfected with vectors encoding the indicated constructs. The next day, cells were treated with MG-132 (10 µM), P5091 (20 µM) as indicated or DMSO as a control for 6 h before lysis. Lysates were subjected to pulldown with Ni–NTA-agarose. Pulldowns and lysates were analyzed by immunoblotting with the indicated antibodies. (**A**, **B**) Western blots from three independent experiments were analyzed with ImageJ, the fold change in ubiquitinated GFP-DLC1 species was determined by normalizing their signal intensity to that of control sample, and is presented as mean ± s.d. Full-length western blots are provided as Supplementary Information.

The observed effects on DLC1 protein levels are in line with the hypothesis of HECTD1 and USP7 being positive and negative regulators of DLC1 ubiquitination, respectively. To test this, we overexpressed His-Ub and GFP-tagged DLC1 together with USP7 or HECTD1 in cells and performed pulldowns with Ni–NTA-agarose to enrich for ubiquitinated proteins. Using SDS-PAGE and immunoblotting analysis higher migrating polyubiquitinated GFP-DLC1 species could be detected in the pulldown (Fig. 3A). Co-expression of HECTD1 led to increased DLC1 signals while co-expression of USP7 diminished DLC1 ubiquitination in HEK293T cells. Conversely, more polyubiquitinated DLC1 species could be detected upon inhibition of USP7 in MCF7 cells (Fig. 3B). However, using different strategies of enrichment for ubiquitinated proteins combined with inhibition of the proteasome and deubiquitinating enzymes we could not conclusively show ubiquitination of endogenous DLC1, similar to a previous report^18^. This might suggest that polyubiquitinated DLC1 species are extremely low abundant. We also cannot exclude that attached ubiquitin moieties preclude detection in immunoblots or immunoprecipitation by monoclonal anti-DLC1 antibodies.

**Figure 4:**
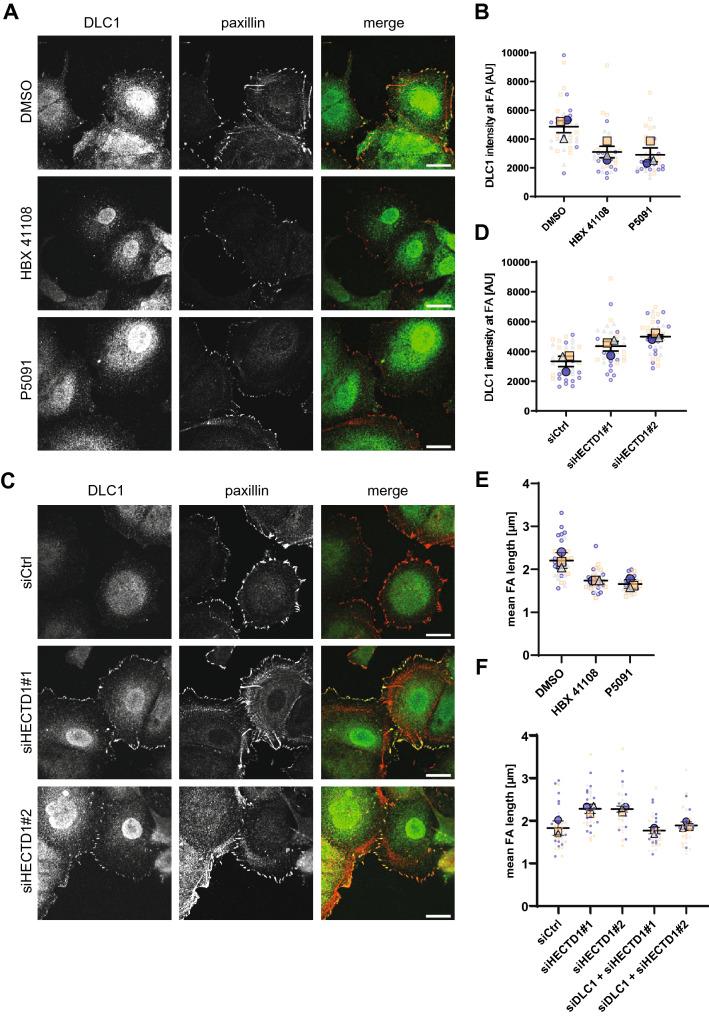
HECTD1 and USP7 regulate DLC1 levels at focal adhesions. (**A**) MCF7 cells were transfected with the indicated siRNAs, seeded on collagen-coated glass coverslips and fixed 72 h later. (**C**) MCF7 cells were seeded on collagen-coated glass coverslips. The next day, cells were treated with P5091 (20 µM), HBX 41108 (10 µM) or DMSO as a control for 6 h before fixation. (**A**,**C**) Fixed cells were stained with DLC1- and paxillin-specific primary antibodies, followed by AlexaFluor488 (green)-coupled and AlexaFluor546 (red)-coupled secondary antibodies, respectively. Images show a single basal section, scale bar: 20 µm. (**B**, **D**) The mean intensity of the DLC1 signal at focal adhesions over the whole image was quantified using ImageJ. N = 36 images, n = 3. Statistical comparison of means by RM-ANOVA with Dunnett’s multiple comparison test: (**B**) DMSO vs. HBX41108: *p* = 0.0252; DMSO vs. P5091: *p* = 0.0178. (**D**) siCtrl vs. siHECTD1#1: *p* = 0.0171; siCtrl vs. siHECTD1#2: *p* = 0.0029. (**E**) Mean focal adhesion length per cell in samples from (**A**) was analyzed using ImageJ. N = 36, 31, 30; n = 3. Statistical comparison of means by RM-ANOVA with Dunnett’s multiple comparison test: DMSO vs. HBX 41108: *p* = 0.0064; DMSO vs. P5091: *p* = 0.0036. (**F**) MCF7 cells were transfected with the indicated siRNAs. After 2 days, cells were seeded on collagen-coated glass coverslips and fixed 16 h later. Cells were stained with paxillin-specific primary antibody followed by AlexaFluor488-coupled secondary antibody. Mean focal adhesion length per cell was analyzed using ImageJ. N = 40, 36, 29, 35, 30; n = 3. Statistical comparison of means by 1-way ANOVA with Bonferroni’s multiple comparison test: siCtrl vs siHECTD1#1: *p* = 0.0017; siCtrl vs. siHECTD1#2: *p* = 0.002; siHECTD1#1 vs. siDLC1 + siHECTD1#1: *p* = 0.0007; siHECTD1#2 vs. siDLC1 + siHECTD1#2: *p* = 0.0056.

Through binding of tensins, talin or FAK, active DLC1 localizes to FAs^10,26,27^, where it is involved in cellular processes such as FA remodeling and mechanotransduction^28,29^. While the recruitment to FAs is fairly well understood, not much is known about the possibility and mechanisms of DLC1 recycling into the cytoplasmic pool during FA turnover or whether DLC1 might be directly degraded after FA disassembly. To investigate if the observed regulation of DLC1 protein levels through HECTD1 or USP7 was also reflected by the DLC1 abundance at FAs, MCF7 cells were stained for endogenous DLC1 after HECTD1 depletion or USP7 inhibition and analyzed by immunofluorescence microscopy. At FA areas marked by paxillin, the mean intensity of the DLC1 signal was decreased after treatment with USP7 inhibitors (Fig. 4A,B), whereas HECTD1 knockdown led to a significant increase in abundance of DLC1 (Fig. 4C,D). DLC1 was previously implicated in focal adhesion turnover whereby DLC1 depletion promoted the accumulation of smaller paxillin-positive adhesive structures^28,30^. In line with these reports, lower DLC1 abundance at FAs upon USP7 inhibition also resulted in a decrease in mean FA length (Fig. 4E). Conversely, we observed an increase in FA length after HECTD1 knockdown which was abolished after simultaneous depletion of DLC1 (Fig. 4F). While these results do not reveal whether ubiquitination of DLC1 is mediated at FAs or elsewhere, they suggest that the local alteration of DLC1 protein levels impact adhesion signaling and FA functions.

## Discussion

In this study, we discovered new regulators of DLC1 proteostasis in breast cancer cell lines. Previously, only the CRL4A-DDB1-FBXW5 RING-type ubiquitin ligase complex was described to regulate DLC1 levels in NSCLC and has recently also been implicated in DLC1 regulation in mesenchymal stem cells^18,31^. Notably, in our list of putative DLC1 interaction partners obtained by mass spectrometry we identified another cullin-RING ligase substrate receptor, DCAF7. It will be interesting to study the potential crosstalk of these degradation pathways in various cancer entities and also non-transformed cellular systems, to additionally understand their role in basal DLC1 turnover. Further, it remains to be investigated if DLC1 is a direct substrate of HECTD1 or whether the regulation of DLC1 turnover occurs indirectly, for example in a complex with the scaffolding protein IQGAP1^32,33^, and whether HECTD1 might further play a role in transcriptional regulation of DLC1.

In NSCLC, targeting the mediators of DLC1 degradation restored DLC1-dependent growth suppression ^18^. HECTD1 was already previously implicated in the regulation of adhesive structures, whereby HECTD1 depletion inhibited cell migration and invasion^23^. In contrasting reports, homozygous inactivation of HECTD1 in mouse embryonic fibroblasts and HECTD1 depletion in breast cancer cells was associated with increased cell motility and epithelial-to-mesenchymal transition^32,34^. However, these observations differ in the methods used to manipulate HECTD1 from knockdown by siRNA or shRNA to homozygous inactivating mutation. To better understand the implications of the HECTD1 and DLC1 interplay for cell adhesion, cell migration and epithelial-to-mesenchymal transition, tools that enable acute perturbations like a specific HECTD1 inhibitor or optogenetic approaches may provide further insights. This might also help to shed light on the subcellular localizations at which regulation of DLC1 degradation takes place. Interestingly, a recent report proposed oncogenic functions for nuclear DLC1 in melanoma^35^. Here, it would be especially interesting to study whether nuclear DLC1 escapes proteasomal degradation and whether USP7, which is enriched in the nucleus, plays a specific role in this setting. Inhibition of USP7 has emerged as a potential therapeutic approach to restore p53 expression in various cancers^24^, additional degradation of potentially oncogenic DLC1 might be beneficial. The USP7 inhibitors used in our study were shown to employ different modes of action: HBX 41108 acts in an allosteric and reversible manner^36^, while analogs of P5091 covalently bind to the catalytic centre^37^. Nevertheless, the possibility of off-target effects contributing to the observed downregulation of DLC1 cannot be fully excluded. Besides deciphering the molecular mechanisms of DLC1 ubiquitination and deubiquitination, it will be crucial to distinguish direct effects of USP7 on DLC1 from secondary effects upon longer-term USP7 inhibition to understand the collective contribution of different factors to the disruption of DLC1 proteostasis during carcinogenesis. For example, p53 was implicated in DLC1 transcription^5,38^ whereas another USP7 substrate, PTEN^39^, was shown to regulate DLC1 subcellular localization^40^. In the TNBC cell lines expressing high levels of DLC1, phosphorylation might be another means leading to the functional inactivation of its RhoGAP activity^13–15^.

## Methods

### Antibodies and reagents

The following antibodies were used in this study: mouse anti-Flag mAb (used 1:1000 in WB, F1804), rabbit anti-GAPDH mAb (used 1:10000 in WB) (G9545), mouse anti-α-tubulin mAb (used 1:10000 in WB, 05–829) and mouse anti-vinculin (used 1:500 in WB, V9131) from Sigma-Aldrich (St. Louis, MO, USA); mouse anti-DLC1 mAb (used 1:500 in WB and IF, 612021) and mouse anti-FAK mAb (used 1:1000 in WB, 610088) from BD Biosciences (Heidelberg, Germany); rabbit anti-paxillin pAb (used 1:500 in WB and IF, sc-5574) and mouse anti-His mAb (used 1:200 in WB, sc-8036) from Santa Cruz Biotechnology (Dallas, TX, USA); rabbit anti-USP7 mAb (WB 1:1000, 4833), rabbit anti-GFP mAb (WB 1:1000, 2956) and rabbit anti-HA mAb (WB 1:1000, 3724) were from Cell Signaling (Danvers, MA, USA); rabbit anti-HECTD1 pAb (WB 1:1000, 20605–1-AP) was from Proteintech (Manchester, UK). HRP-labeled secondary anti-mouse and anti-rabbit IgG antibodies were purchased from and Dianova (Hamburg, Germany), Alexa-Fluor®-labeled secondary IgG antibodies were from Invitrogen (Carlsbad, CA, United States). Inhibitors used were: Bortezomib from UBPBio (Dallas, TX), MG-132 from Selleck Chemicals (Houston, TX); PR-619, HBX 41108 and P5091 from Cayman Chemicals (Ann Arbor, MI); cycloheximide from Santa Cruz (Dallas, TX).

### Cell culture and transfection

All cells were incubated in a humidified atmosphere of 5% CO_2_ at 37 °C. BT-20 (obtained from CLS Cell Lines Service GmbH, Eppelheim, Germany) were cultivated in DMEM/F12 medium (Invitrogen) supplemented with 5% fetal calf serum (FCS; PAA Laboratories, Cölbe, Germany). BT-474 (kindly provided by Nancy Hynes, FMI, Basel, Switzerland), BT-549 (CLS), HCC1806 (ATCC, Manassas, USA), Hs 578 T (kindly provided by Bernhard Lüscher, RWTH Aachen, Germany), MDA-MB-231 (CLS), MDA-MB-436 (kindly provided by Institute of Clinical Pharmacology, Stuttgart, Germany), MDA-MD-453 (kindly provided by Jane Visvader, Walter and Eliza Hall Institute of Medical Research, Melbourne, Australia), MDA-MB-468 (CLS), SUM159PT (obtained from the DKFZ, Heidelberg, Germany) were cultured in DMEM (Invitrogen) supplemented with 10% FCS. HEK293T (ATCC), MCF7 (kindly provided by Cornelius Knabbe, Institute of Clinical Pharmacology, Stuttgart, Germany), SKBR3 (CLS), T-47D, ZR-75-1 and ZR-75-30 (kindly provided by Bernhard Lüscher, RWTH Aachen, Germany) were cultured in RPMI 1640 supplemented with 10% FCS. Breast cancer subtypes were grouped according to^41^. For plasmid transfection of MCF7 cells, Lipofectamine LTX with Plus Reagent (Invitrogen) was used according to manufacturer’s instructions. Plasmid transfection of HEK293T cells was performed using a 1:3 (w/w) mixture of DNA to polyethylenimine (Sigma Aldrich). pEGFPN1-DLC1 was generated by PCR amplification using FLAG-DLC1^13^ as a template with the primers: 5’- CGCGGATCCACCATGTGCAGAAAGAAGCCGGACACC-3’ and 5’- CGCGGATCCCTAGATTTGGTGTCTTTGGTTTC-3’. The PCR product was cloned into the pEGFP-N1 vector (Clontech) via BamHI. His-ubiquitin plasmid (pMT107) was a gift of Reinhard Fässler (MPI of Biochemistry, Martinsried, Germany). pCI-neo Flag HAUSP (Flag-USP7) was from Bert Vogelstein (Addgene plasmid #16655)^42^. pCMV-HA-HECTD1 was a gift of Irene Zohn (Center for Genetic Medicine Research, Children’s National, Washington, DC)^43^. For RNAi, cells were transfected with siRNA for 72 h using Lipofectamine RNAiMAX (Invitrogen) according to manufacturer’s instructions. The siRNAs used were: negative control siRNA (siCtrl, ON-TARGETplus® non-targeting control pool D-001810–10; Dharmacon, Lafayette, CO), siHECTD1#1 (Silencer® Select HECTD1 s24575; ambion life technologies), siHECTD1#2 (Silencer® Select HECTD1 s24576; ambion life technologies), custom designed Silencer® Select human DLC1 siRNA (siDLC1, s530697, ambion life technologies), siUSP7#1 (Silencer® Select USP7 s15439; ambion life technologies), siUSP7#2 (Silencer® Select USP7 s15440; ambion life technologies).

### Cell lysis and immunoblotting

Cells were lysed in RIPA buffer [50 mM Tris (pH 7.5), 150 mM NaCl, 1% Triton-X-100, 0.5% sodium deoxycholate, 1 mM EDTA, 0.5 mM PMSF, 0.1% SDS, 1 mM sodium orthovanadate, 10 mM sodium fluoride, and 20 mM β-glycerophosphate plus Complete protease inhibitors without EDTA (Roche)] and lysates were clarified by centrifugation (16,000 × g, 10 min). Protein concentration was determined by Bio-Rad DC protein assay. Proteins were separated by SDS-PAGE and transferred to polyvinylidene difluoride membranes (Roth, Karlsruhe, Germany). Alternatively, lysates were loaded on 4–12% NuPAGE® Novex Bis–Tris gels (Invitrogen) and transferred to nitrocellulose membranes (iBlot®Gel Transfer Stacks; Invitrogen). Membranes were blocked with 0.5% blocking reagent (Roche) in PBS containing 0.05% Tween-20 and incubated with primary antibodies, followed by HRP-labeled secondary antibodies for ECL-based (Pierce, Rockford, IL) visualization with the Amersham600 system (GE Healthcare) or the Fusion Solo (VilberLourmat). Original western blots of all cropped blots are provided as Supplementary Information.

### Quantitative real-time PCR

RNA was isolated from cells using the NucleoSpin RNA kit (Macherey–Nagel) according to manufacturer’s instructions. 100 ng RNA were used for real-time PCR, using the Power SYBR® Green RNA-to-CT 1-Step kit (Thermo Fisher) with the following primers: DLC1-F: tgaagatttcctgttccccatc, DLC1-R: agtatttagacgcctgcatagag, HECTD1-F: ATTGCTGGAATGGCTACAGATG, HECTD1-R: AAGGGCTGG TAAGAAAGTGCG, RPLP0-F: CTCTGCATTCTCGCTTCCTGG AG, RPLP0-R: CAGATGGATCAGCCAAGAAGG. Analysis was performed using the CFX96 Touch Real-Time PCR Detection System (Bio-RAD). To analyze the fold change gene expression, the double delta Ct analysis was used (fold change = 2(-ΔΔCt)). RPLP0 served as control gene.

### NanoLC-MS/MS analysis and MS data processing

MCF7 cells were transfected with constructs encoding GFP-tagged DLC1 or GFP as a control and lysed in 1% TEB buffer (RIPA buffer without sodium deoxycholate and SDS). GFP proteins were immunoprecipitated using GFP-Trap agarose beads (Chromotek, Martinsried, Germany). Beads were washed with 1% TEB and PBS followed by elution with 0.1 M glycine (pH 2.5) and neutralization with 1/10 volume of 1 M Tris (pH 8.0). Protein expression and immunopurification were verified by parallel immunoblotting.

Proteins were purified on a NuPAGE 12% gel (Invitrogen) and Coomassie-stained gel pieces were digested in gel with trypsin as described previously^44^. After desalting using C18 stage tips^45^ peptide mixtures were run on an EasyLC nano-HPLC coupled to an LTQ Orbitrap Elite mass spectrometer (both Thermo Fisher Scientific) as described elsewhere^46^ with slight modifications: the peptide mixtures were separated using a 127 min segmented gradient from 10-33-50-90% of HPLC solvent B (80% acetonitrile in 0.1% formic acid) in HPLC solvent A (0.1% formic acid) at a flow rate of 200 nl/min. Precursor ions were acquired in the mass range from m/z 300 to 2000 in the Orbitrap mass analyzer at a resolution of 120,000. Accumulation target value of 106 charges was set. The 15 most intense ions were sequentially isolated and fragmented in the linear ion trap using collision-induced dissociation at the ion accumulation target value of 5000 and default CID settings. Sequenced precursor masses were excluded from further selection for 60 s.

Acquired MS spectra were processed with MaxQuant software package version 1.5.2.8^47^ with integrated Andromeda search engine^48^. Database search was performed against a Homo sapiens database containing 91,675 protein entries, and 285 commonly observed contaminants, plus the GFP-DLC1 sequence. Endoprotease trypsin was defined as protease with a maximum of two missed cleavages. Oxidation of methionine, phosphorylation of serine, threonine and tyrosine, methylation on lysine and arginine residues, acetylation of lysine and the protein N-terminus were specified as variable modifications. Carbamidomethylation on cysteine was set as fixed modification. Initial precursor mass tolerance was set to 4.5 parts per million, and at the fragment ion level 0.5 Da was set for CID fragmentation. Peptide, protein and modification site identifications were reported at a false discovery rate of 0.01, estimated by the target-decoy approach^49^. The iBAQ algorithm was enabled to estimate quantitative values by dividing the sum of peptide intensities of all detected peptides by the number of theoretically observable peptides of the matched protein^50^.

### Co-immunoprecipitation

The day after transfection, HEK293T cells were lysed in 1% NEB buffer (TEB buffer with Triton-X 100 substituted by NP-40). Lysates were clarified by centrifugation (16,000 × g, 10 min). Equal amounts of protein were diluted with lysis buffer to a final concentration of 1 mg/ml and incubated with specific antibodies for 3 h at 4 °C. Immune complexes were collected using protein G agarose (Thermo Scientific) for 1 h at 4 °C and washed three times with lysis buffer. Alternatively, GST-tagged GFP-nanobody (Addgene plasmid #61838^51^) coupled to glutathione sepharose beads was added to the lysates for 1 h at 4 °C to pulldown GFP-tagged protein.

### His-Ub pulldown

The day after transfection, cells were lysed in denaturing lysis buffer (8 M urea, 0.1 M Na_2_HPO_4_, 0.1 M NaH_2_PO_4_, 0.01 M Tris–HCl (pH 8), 10 mM β-ME, 10 mM imidazole and 1% Triton X-100) and incubated with Ni–NTA-Agarose (Qiagen) overnight at RT. Beads were washed once with a buffer containing 6 M guanidinium-HCl, 0.1 M Na_2_HPO_4_, 0.1 M NaH_2_PO_4_, 0.01 M Tris–HCl (pH 8) and 10 mM β-ME, and twice with a buffer containing 8 M urea, 0.1 M Na_2_HPO_4_, 0.1 M NaH_2_PO_4_, 0.01 M Tris–HCl (pH 8), 10 mM β-ME, 10 mM imidazole before elution by boiling in 5 × SDS-sample buffer (1 M Tris (pH 6.8), 50% glycerol, 0.5 M DTT, 10% SDS) plus 200 mM imidazole.

### Immunofluorescence microscopy and image analysis

For analysis of DLC1 abundance at FAs, cells grown on glass coverslips coated with 10 μg/ml collagen R (Serva, Heidelberg, Germany) were fixed and permeabilized with 4% PFA containing 0.1% Triton X-100 for 10 min at RT. After washes in PBS, cells were incubated for 15 min with 150 mM glycine in PBS and blocking was performed with 5% goat serum (Invitrogen) in PBS containing 0.1% Tween-20 for 30 min at RT. Fixed cells were incubated with DLC1- and paxillin-specific primary antibodies diluted in blocking buffer for 2 h at RT, followed by incubation with AlexaFluor® (488, 546) labeled secondary antibodies in blocking buffer for 1 h at RT. Coverslips were mounted in Fluoromount-G® (SouthernBiotech; Birmingham, AL) and analyzed at RT on a spinning disc Axio Observer Z1/7 microscope (Carl Zeiss, Oberkochen, Germany) equipped with a Plan-Apochromat 63x/1.40 DIC M27 (Carl Zeiss) oil immersion objective and an evolve 512 EMCCD camera using 488-, 561-nm laser excitation. Each set of replicates (12 images each, n = 3) was acquired with the same confocal settings and analyzed using the ImageJ software (NIMH; Bethesda, Maryland). For quantification of DLC1, focal adhesion areas were defined by paxillin staining and mean intensity of the DLC1 signal over the whole image was measured. Data are presented as SuperPlot^52^. For analysis of FA length after siRNA-mediated depletion of HECTD1 alone or simultaneous depletion of DLC1, cells were fixed with 4% PFA for 10 min at RT and permeabilized with 0.2% Triton X-100. Blocking and staining was performed as above. The length of paxillin-positive focal adhesions was determined manually using the ImageJ software.

## Supplementary Information

Supplementary Information 1.

Supplementary Information 2.

## Data Availability

The mass spectrometry proteomics data have been deposited to the ProteomeXchange Consortium via the PRIDE partner repository with the dataset identifier PXD027186.
