# Supplementary material for: Proteasomal turnover of the RhoGAP tumor suppressor DLC1 is regulated by HECTD1 and USP7
Source: Sci Rep. 2022 Mar 23;12:5036. doi: 10.1038/s41598-022-08844-3 (PMC8943137; doi:10.1038/s41598-022-08844-3)
Supplement: Supplementary file 1 — Supplementary Information 1. [file 41598_2022_8844_MOESM1_ESM.pdf]

# Supplementary figures

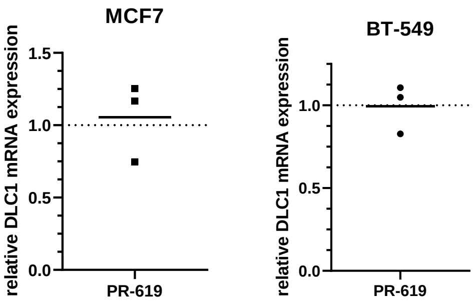


**Supplementary figure S1.** qPCR analysis of DLC1 expression in MCF7 or BT-549 cells after treatment with PR-619 for 6h. Data are presented as mean mRNA expression of treated cells normalized to DMSO control.

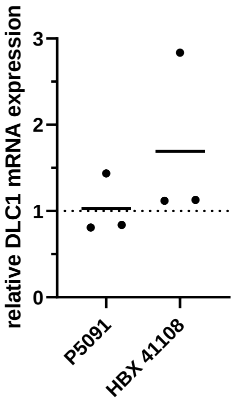


**Supplementary figure S2.** qPCR analysis of DLC1 expression after treatment with P5091 or HBX 41108 for 6h. Data are presented as mean mRNA expression of treated cells normalized to DMSO control.

**Supplementary figures**

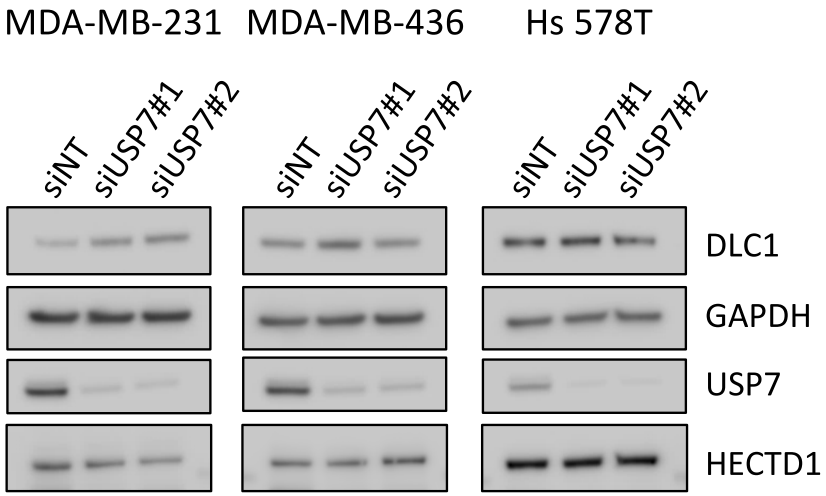


**Supplementary figure S3.** Cells were transfected with control siRNA or two independent siRNAs targeting USP7. 72 h post transfection cells were lysed and lysates were analyzed by immunoblotting with the indicated antibodies.

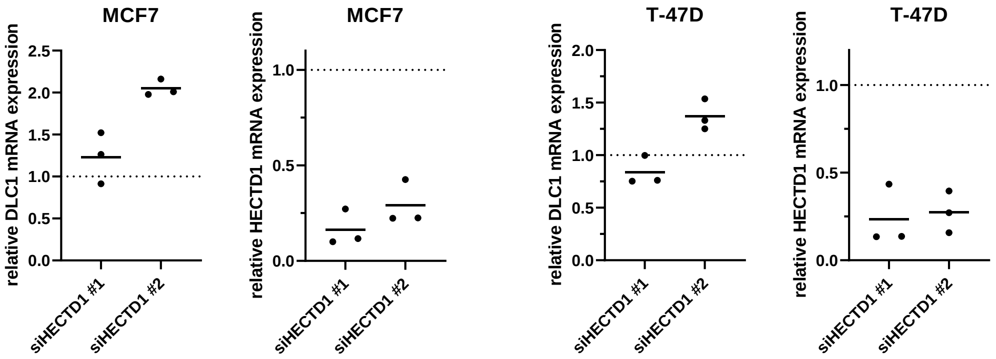


**Supplementary figure S4.** qPCR analysis of DLC1 and HECTD1 expression in MCF7 or T-47D cells 72h after transfection with the indicated siRNAs. Data are presented as mean mRNA expression of treated cells normalized to non-targeting siRNA control.

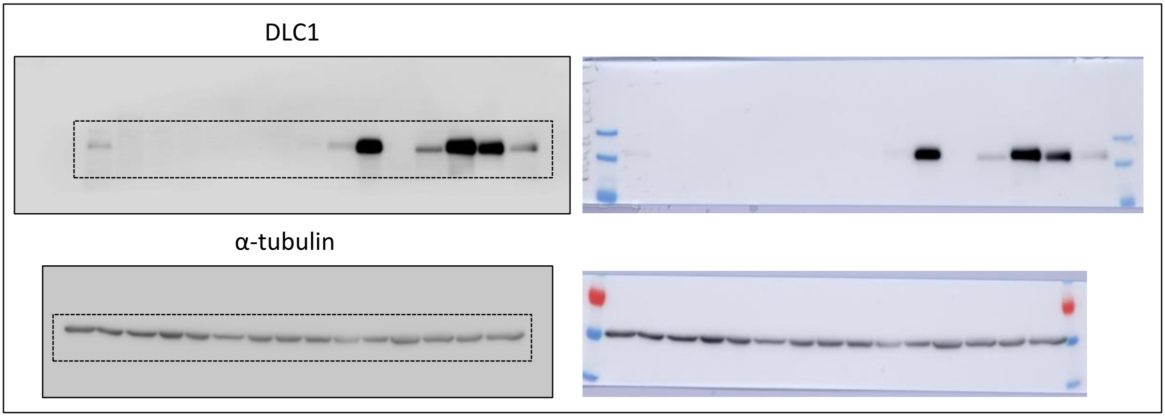


**Supplementary figure S5.** Original western blots corresponding to Figure 1A. Membrane was cut horizontally above ~70 kDa before probing with antibodies

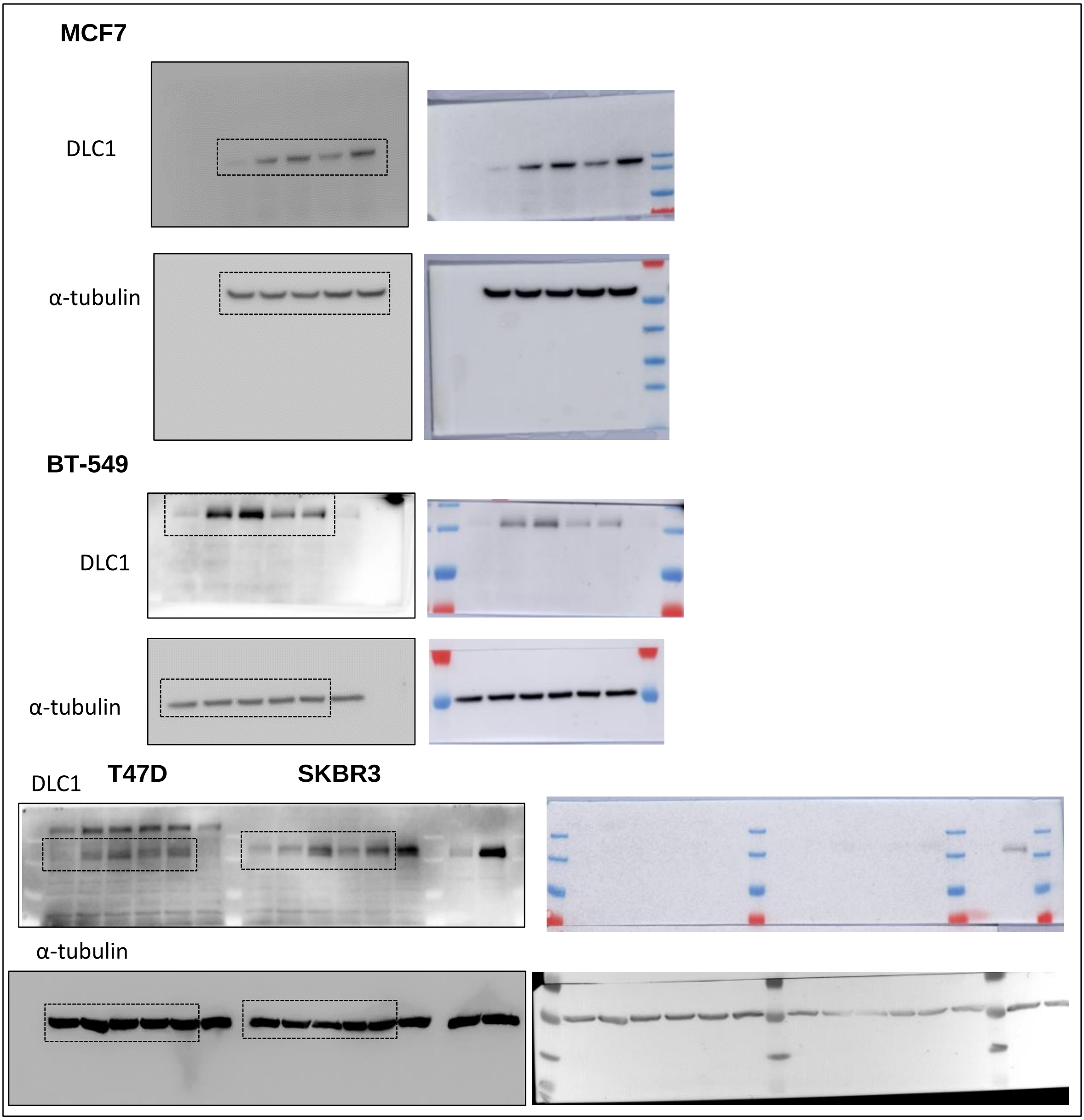


**Supplementary figure S6.** Original western blots corresponding to Figure 1B. Membranes were cut horizontally at ~70 kDa before probing with antibodies.

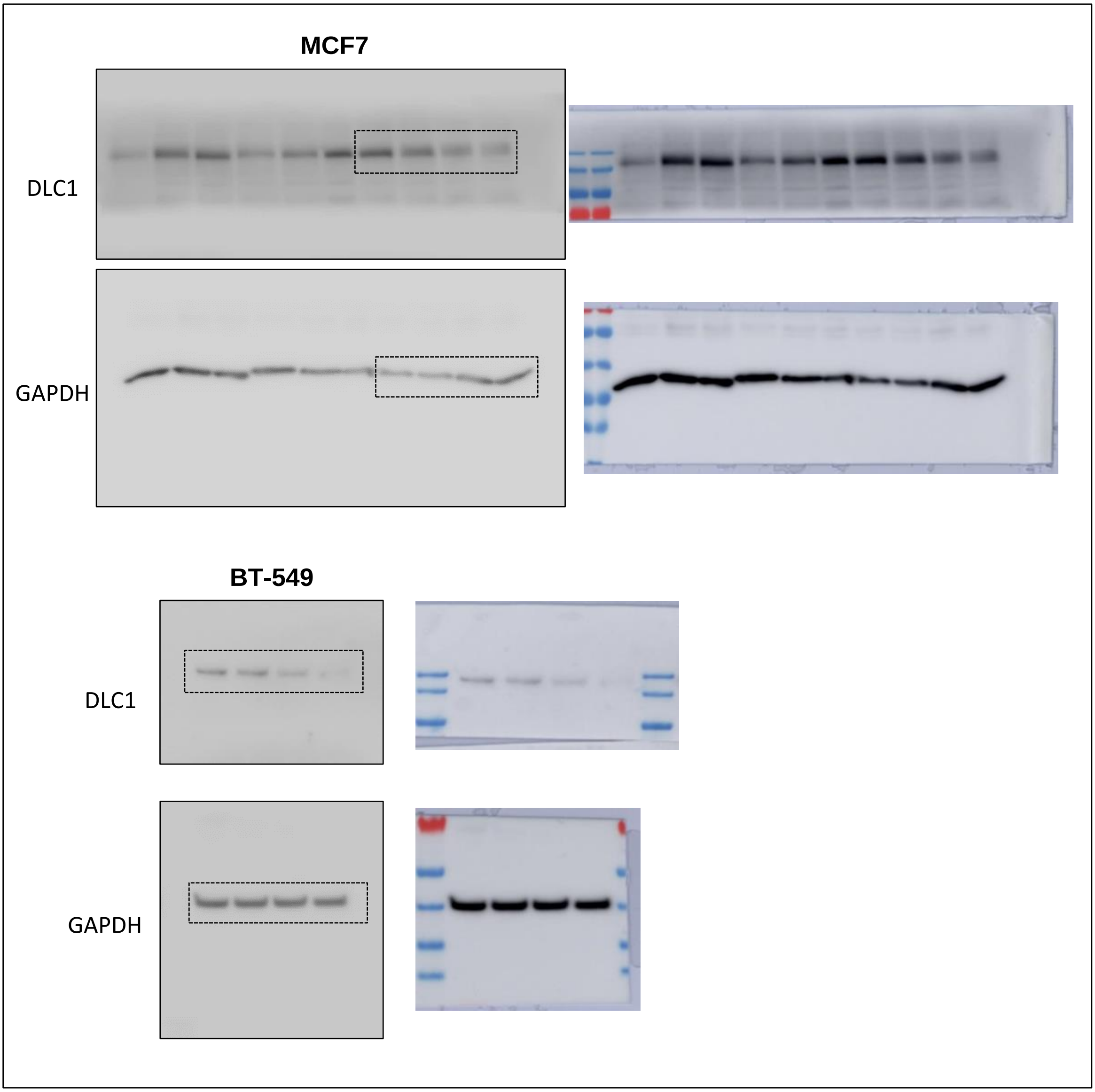


**Supplementary figure S7.** Original western blots corresponding to Figure 1C. Membrane was cut horizontally above ~70kDa before probing with antibodies.

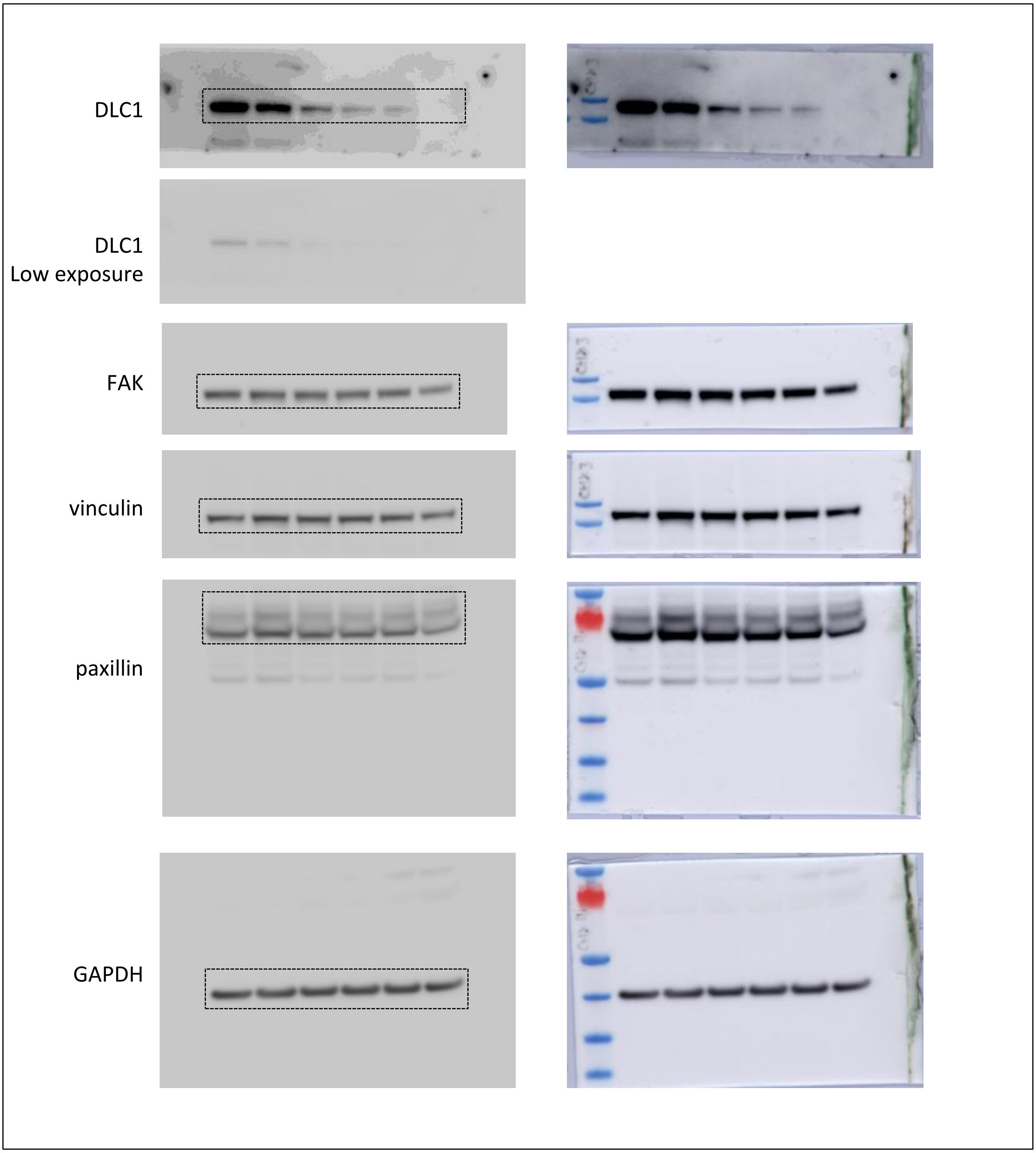


**Supplementary figure S8.** Original western blots corresponding to Figure 1D. Membrane was cut horizontally at ~80kDa before probing with antibodies. Upper part was first probed for DLC1, then reprobed for FAK, then reprobed for vinculin. Lower part was first probed for paxillin, then reprobed for GAPDH.

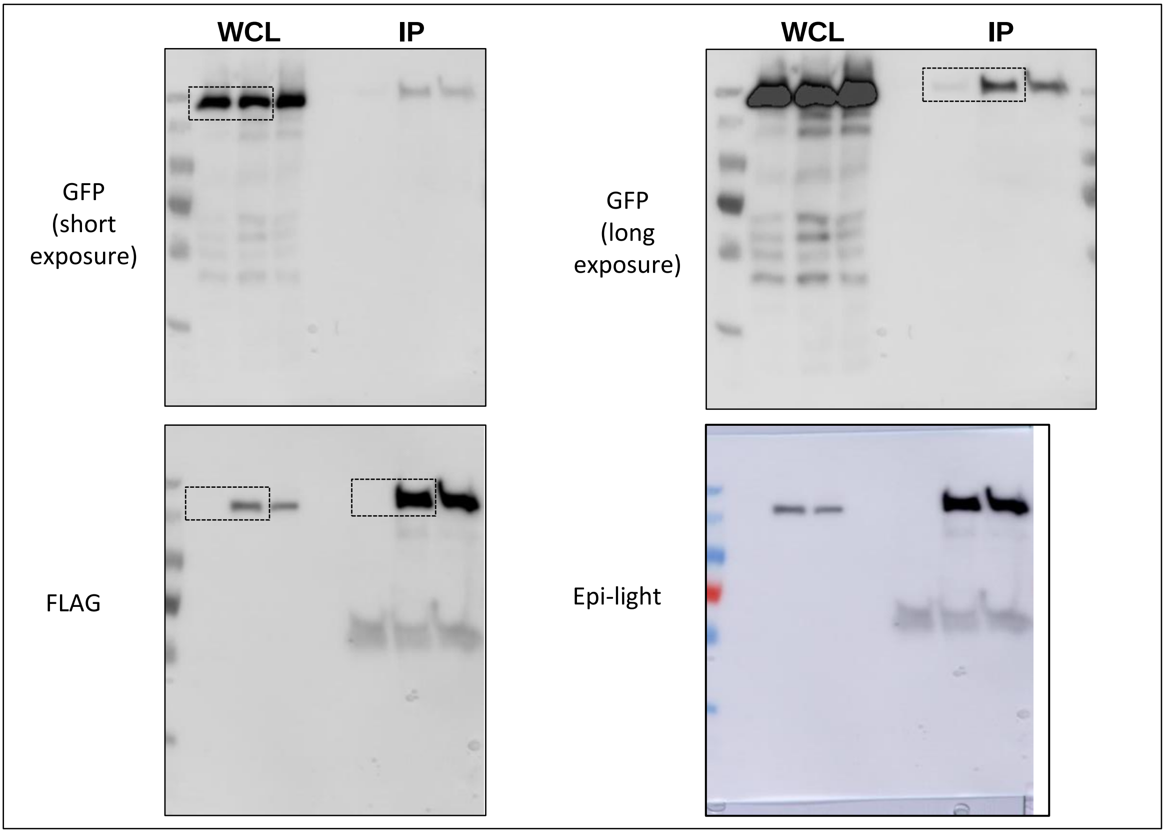


**Supplementary figure S9.** Original western blots corresponding to Figure 2A. Membrane was first probed for GFP, then reprobed for FLAG.

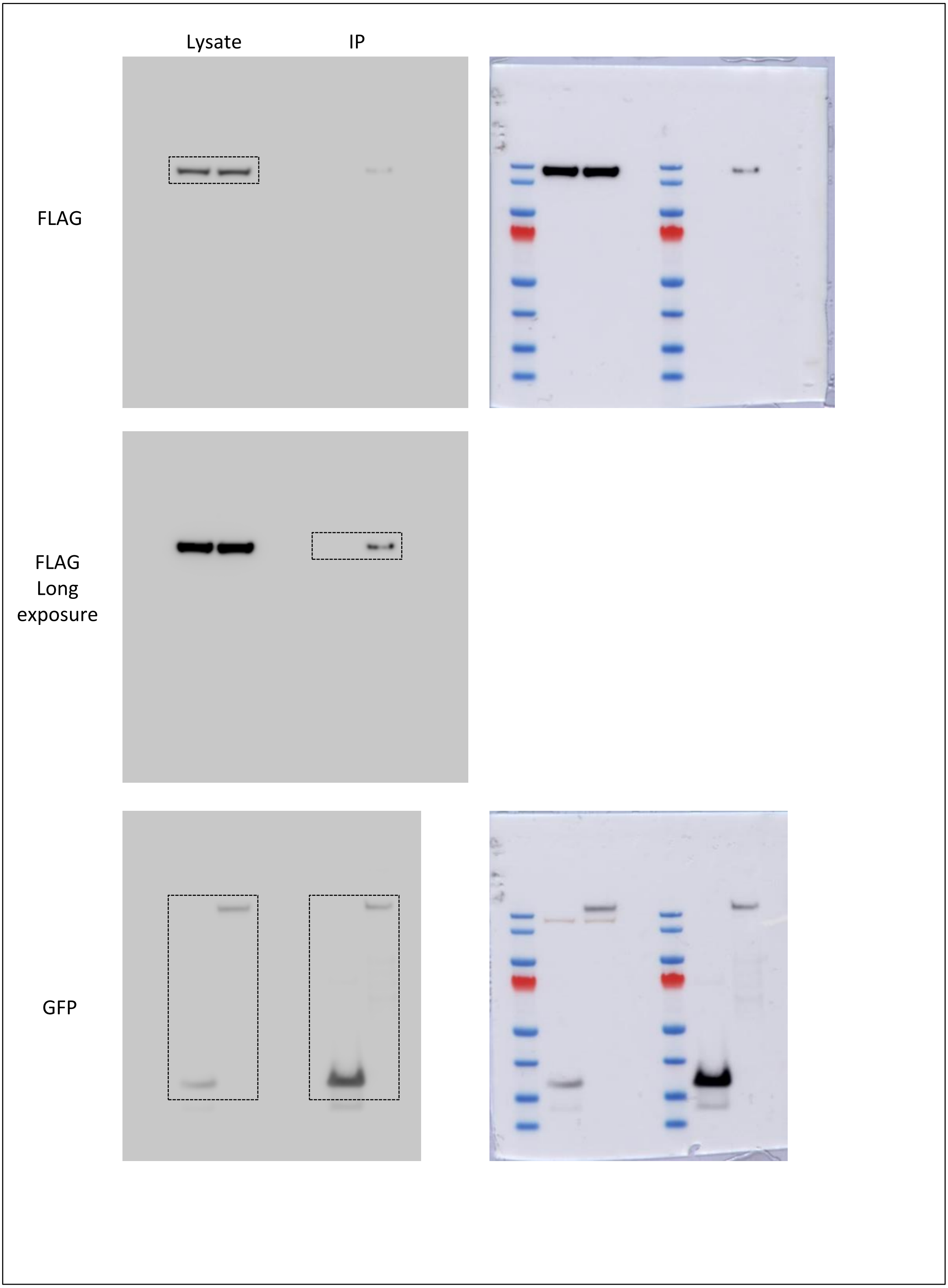


**Supplementary figure S10.** Original western blots corresponding to Figure 2B. Membrane was first probed for FLAG, then reprobed for GFP.

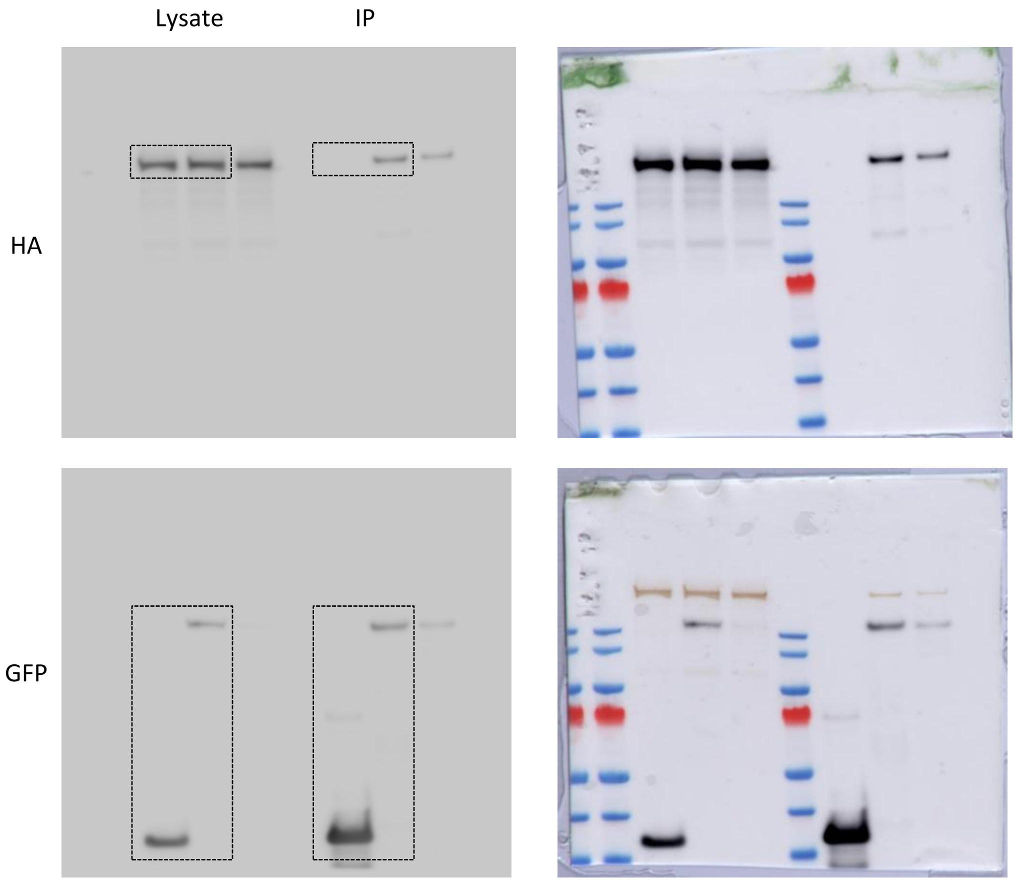


**Supplementary figure S11.** Original western blots corresponding to Figure 2C. Membrane was first probed for HA, then reprobed for GFP.

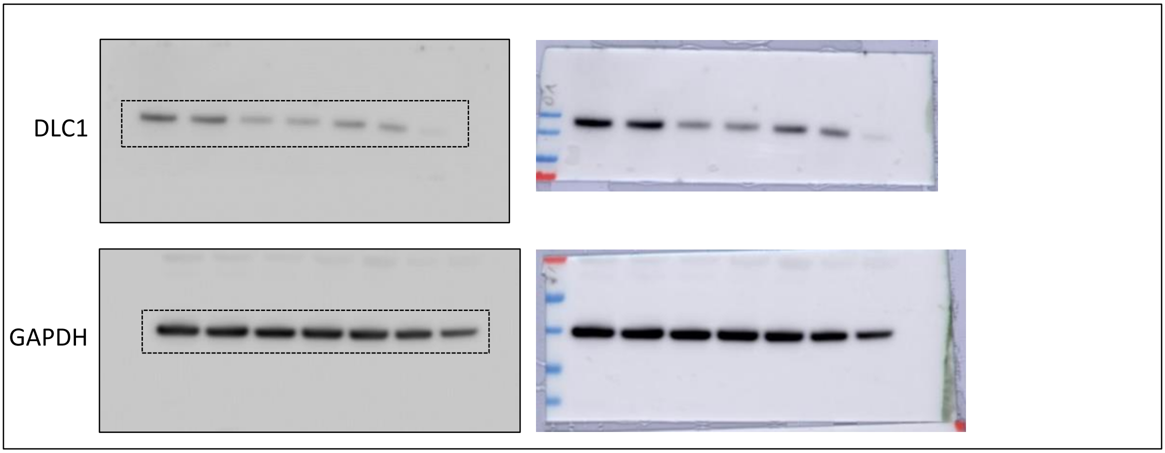


**Supplementary figure S12.** Original western blots corresponding to Figure 2D. Membrane was cut horizontally at ~70 kDa before probing with antibodies.

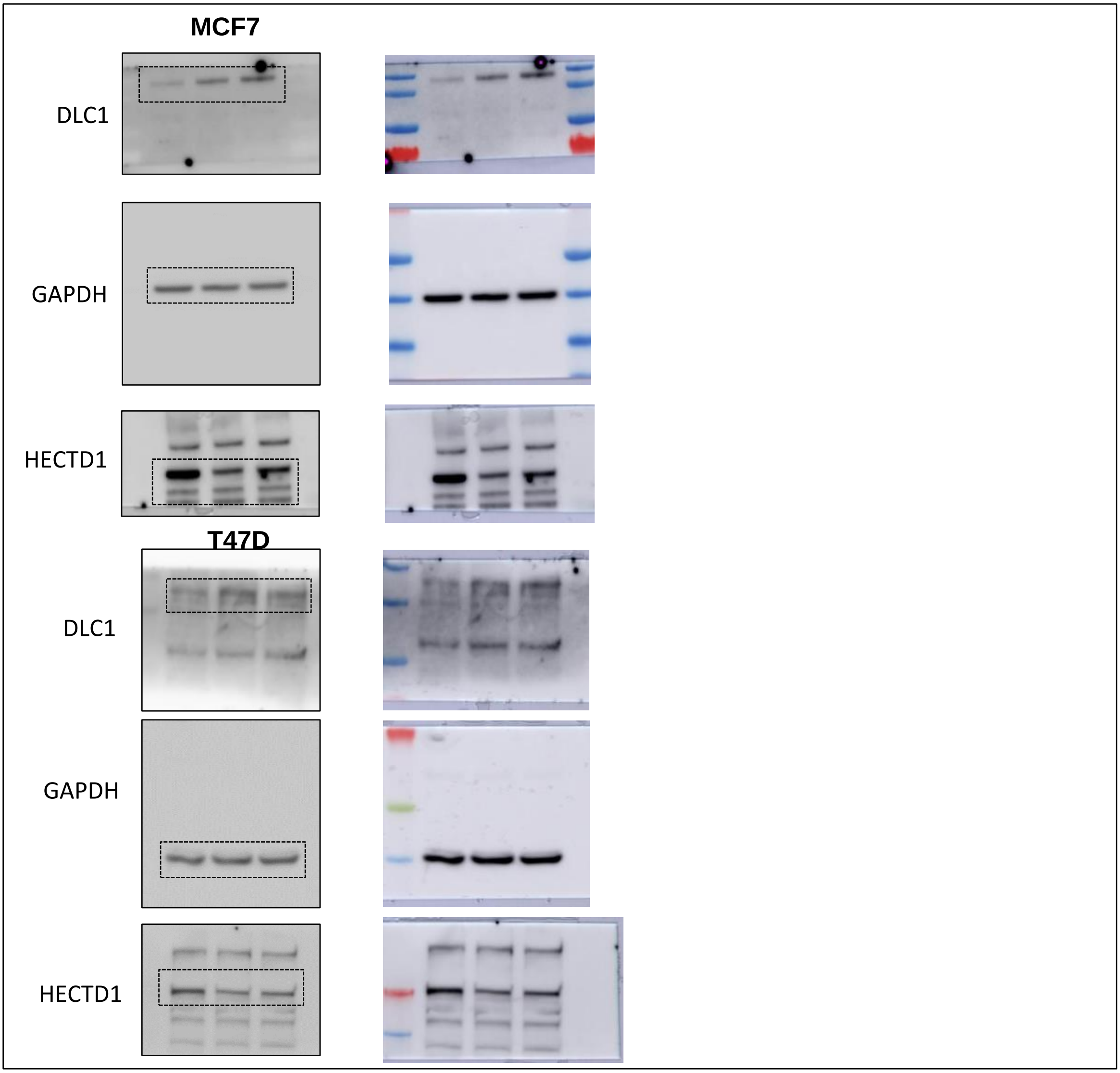


**Supplementary figure S13.** Original western blots corresponding to Figure 2E. Membranes were cut horizontally at ~70 kDa and above ~170 kDa before probing with antibodies

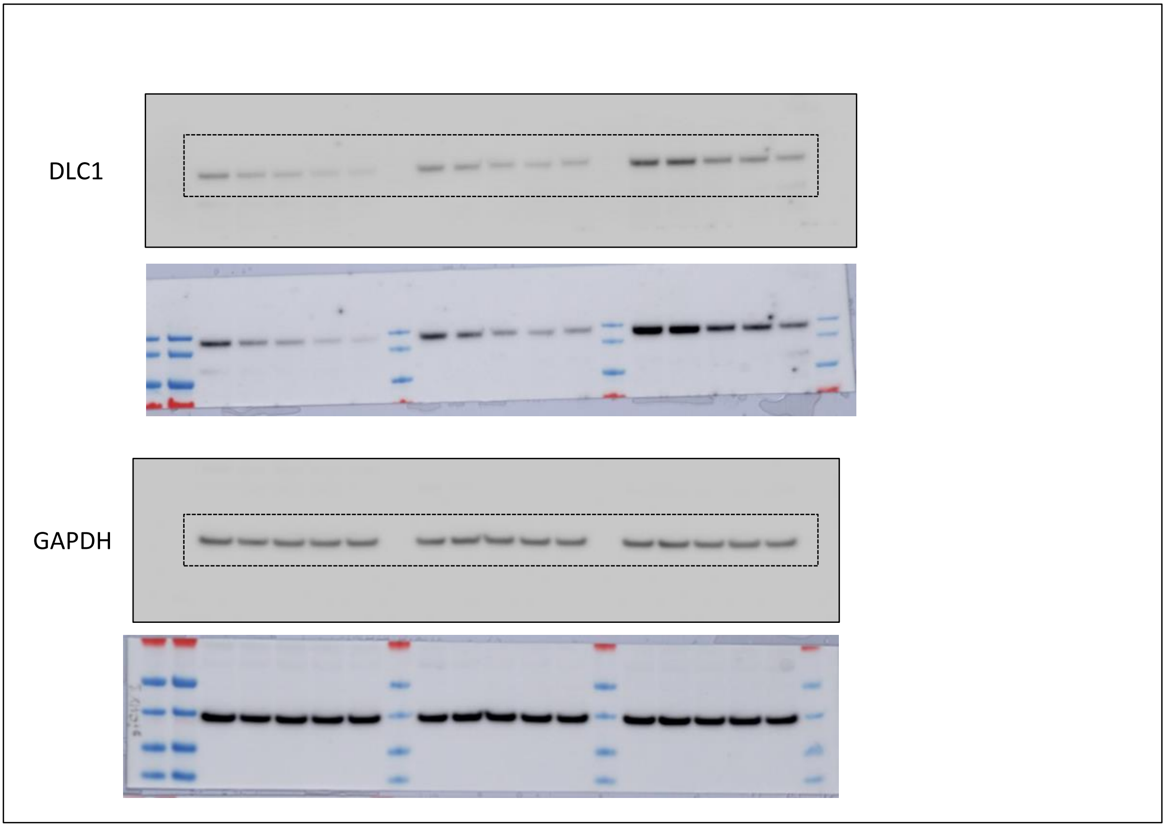


**Supplementary figure S14.** Original western blots corresponding to Figure 2F.
Membrane was cut horizontally at ~70 kDa before probing with antibodies

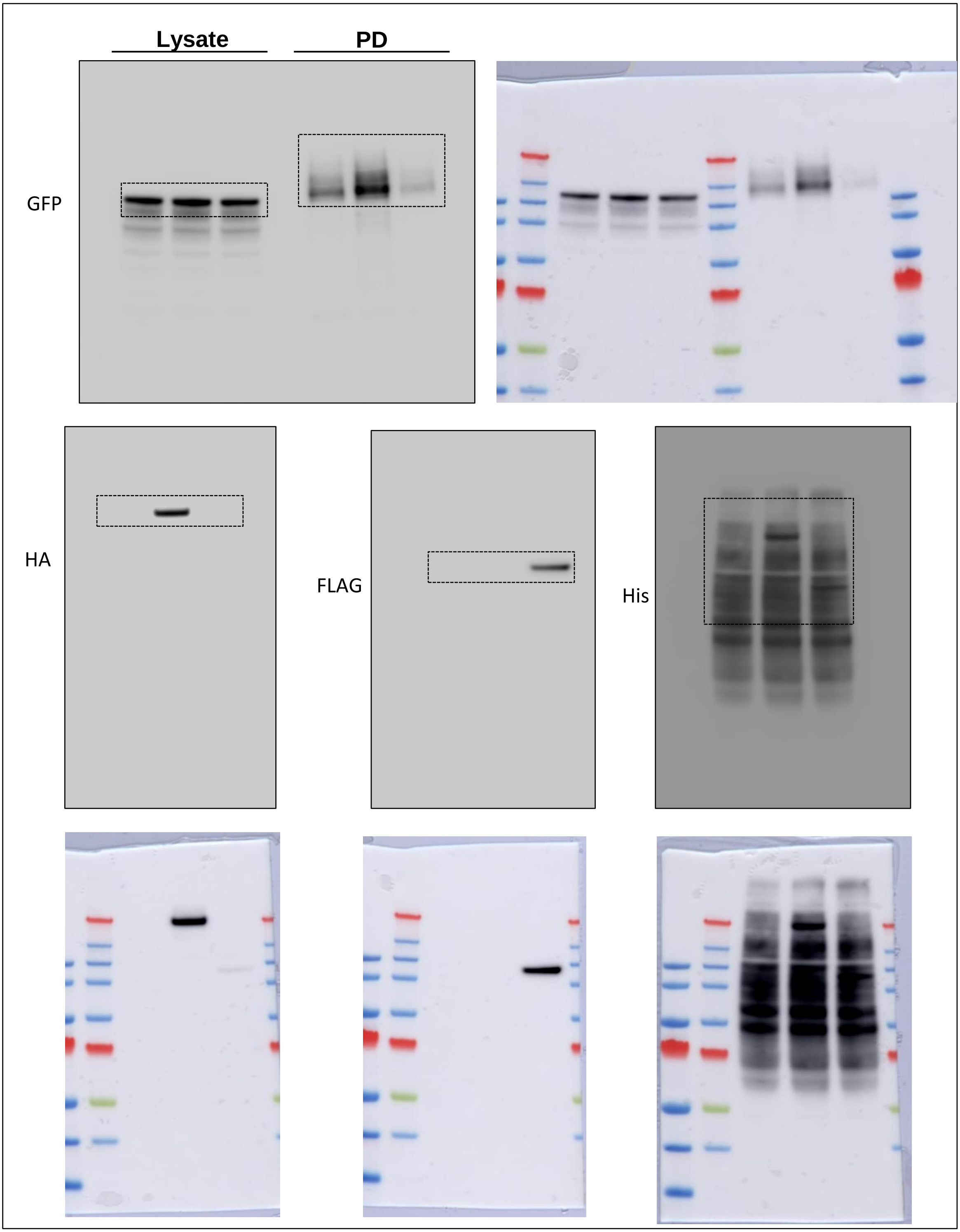


**Supplementary figure S15.** Original western blots corresponding to Figure 3A. Membrane was first probed for GFP, Lysate part was then reprobed for FLAG, HA, and His.

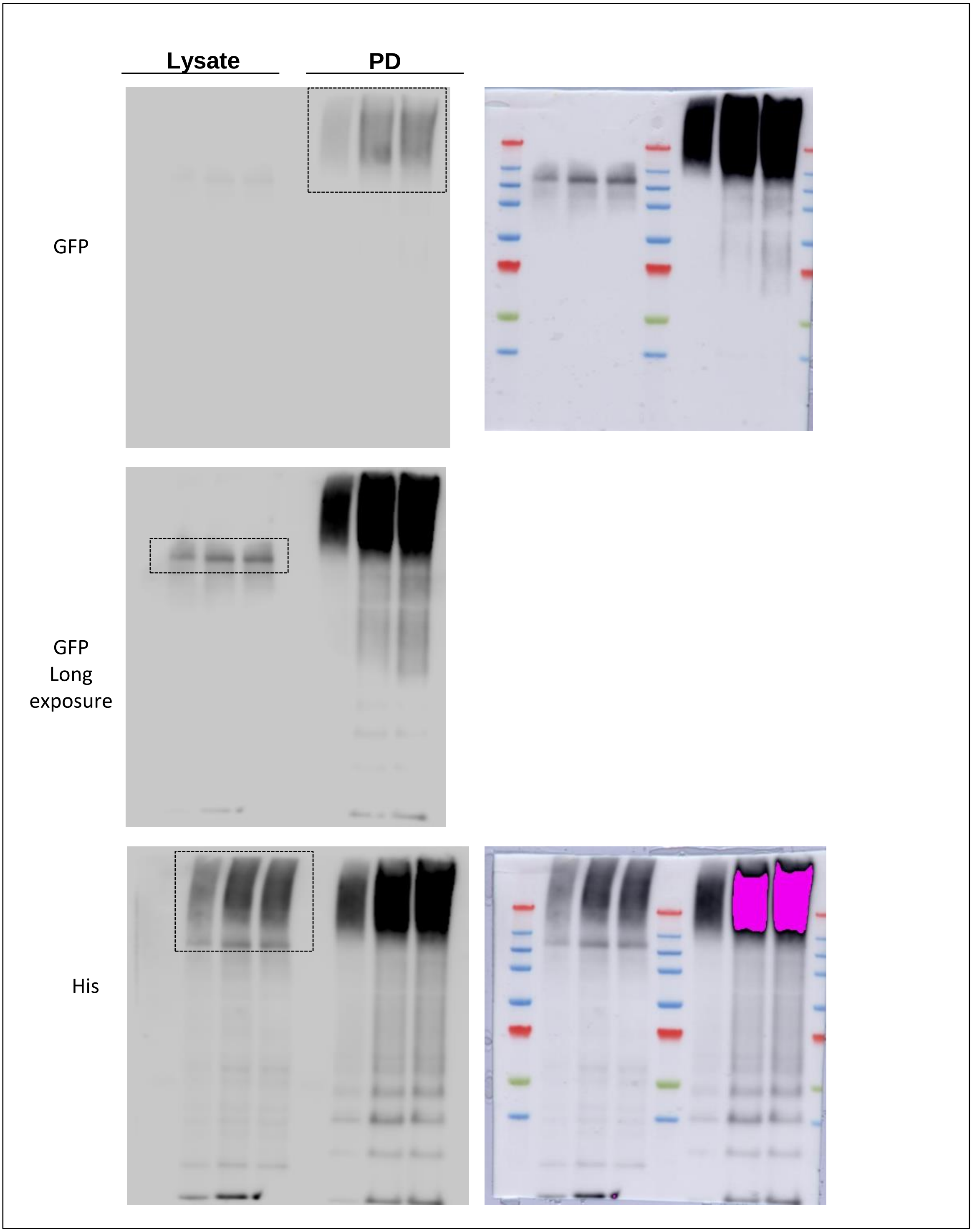


**Supplementary figure S16.** Original western blots corresponding to Figure 3B. Membrane was first probed for GFP, then reprobed for His.
